# Genetics and Genomics of African Rice (*Oryza glaberrima* Steud) Domestication

**DOI:** 10.1186/s12284-020-00449-6

**Published:** 2021-01-07

**Authors:** Peterson W. Wambugu, Marie-Noelle Ndjiondjop, Robert Henry

**Affiliations:** 1 Kenya Agricultural and Livestock Research Organization, Genetic Resources Research Institute, P.O. Box 30148, Nairobi, 00100 Kenya; 2 M’bé Research Station, Africa Rice Center (AfricaRice), 01 B.P. 2551 Bouaké 01, Côte d’Ivoire; 3 Queensland Alliance for Agriculture and Food Innovation, University of Queensland, Brisbane, QLD 4072 Australia

**Keywords:** African rice, *Oryza glaberrima*, Genomics, Domestication, *Oryza barthii* and domestication gene

## Abstract

African rice (*Oryza glaberrima* Steud) is one of the two independently domesticated rice species, the other one being Asian rice (*Oryza sativa* L.). Despite major progress being made in understanding the evolutionary and domestication history of African rice, key outstanding issues remain controversial. There appears to be an underlying difficulty in identifying the domestication centre and number of times the crop has been domesticated. Advances in genomics have provided unprecedented opportunities for understanding the genetic architecture of domestication related traits. For most of the domestication traits, the underlying genes and mutations have been identified. Comparative analysis of domestication genes between Asian and African rice has revealed that the two species went through an independent but convergent evolution process. The genetic and developmental basis of some of the domestic traits are conserved not only between Asian and African rice but also with other domesticated crop species. Analysis of genome data and its interpretation is emerging as a major challenge facing studies of domestication in African rice as key studies continue giving contradictory findings and conclusions. Insights obtained on the domestication of this species are vital for guiding crop improvement efforts.

## Background

Plant domestication is a key evolutionary event where humans modify plant characteristics genetically by selecting for some preferred and favourable traits. Domestication has had a huge impact on food and nutrition security as it involves changes in important traits affecting productivity, adaptability and diverse quality aspects. Knowledge of the domestication process is important for guiding crop improvement efforts (Choi et al. 2019; Purugganan 2019). Domestication plays an important role in diversifying food production.

The *Oryza* genus is one of great economic importance in the world due to the role it plays in global food security. The genus is home to two independently domesticated rice species, *O. sativa* and *O. glaberrima*, whose domestication is believed to have taken place in Asia and Africa respectively. Recent archaeological evidence suggests a third domestication event may have occurred in the Amazon (Hilbert et al. 2017). Although *O. glaberrima* is mainly cultivated in West Africa, there is evidence of this species in the Suriname where it is believed to have been distributed by enslaved Africans (van Andel et al. 2016). African rice possesses immense genetic potential in terms of resistance to biotic and abiotic stress and therefore forms a valuable genetic resource for rice improvement (Wambugu et al. 2013). Due to its importance, the origin and evolution of rice has been extensively studied over the past several decades using archaeological, linguistic, isozyme, molecular and morphological evidence (Ammiraju et al. 2008; Andel 2010; Chen et al. 2013; Molina et al. 2011; Sakai et al. 2011; Stein et al. 2018). Despite these studies, knowledge of the origin, evolutionary history and domestication processes of both *O. sativa* and *O. glaberrima* has remained inconclusive, controversial and contradictory. Debate on the origin and domestication history of African rice has however remained less controversial than that of Asian rice.

Compared to Asian rice, the origin and domestication history of African rice remains grossly understudied. However, over the last few years there has been renewed interest in using whole genome data to unravel this complex domestication history (Choi et al. 2019; Cubry et al. 2018; Veltman et al. 2019; Wang et al. 2014). This interest was most likely inspired by emergence of cheap DNA sequencing technologies. Major advances in genetics and genomics have been recorded for African rice and *O. barthii*, its putative progenitor over the last couple of years (Stein et al. 2018; Wambugu and Henry 2018; Wambugu et al. 2019). Whole genome sequences data is currently publicly available for more than 500 individuals of African rice and *O. barthii.* This provides a valuable genomic resource for dissecting the origin and domestication history of this cultivated *Oryza* species. The models, knowledge and insights obtained for Asian rice remain invaluable and continue to guide domestication studies in African rice (Purugganan 2014). In this paper, we review the origin and domestication dynamics of African rice focusing on the recent advances in knowledge. Of particular interest are those advances that have been driven by various technological innovations in genomics particularly in genome sequencing which have been witnessed over the last decade.

## Review

### Origin and Domestication Models of African Rice

Despite a general consensus on some issues associated with the domestication of African rice, there appears to be an underlying complexity and uncertainty in identifying the exact domestication centre. Debate on the number of times and geographical regions where the crop was domesticated also rages on. The foundation for studies on the origin and domestication history of African rice was laid by the pioneering work of Porteres (1962) which was followed about a decade later by that of Harlan (1971). These two authors proposed the centric and non-centric origin theories which are the major theories around which debate on the origin of many crops revolve. Porteres (1962) postulated that African rice was domesticated in a centric manner where the selection was done in a geographically localized region. According to Porteres (1962), the primary centre of origin was in the Niger River Delta before it later diffused to what this author refers to as secondary centres of diversification along the Senegambian coast and in the Guinea highlands. Porteres (1962) identified two sets of traits in African rice; one of which he referred to as “genetically dominant characteristics” which were common in the centre of origin and the other one which he referred to as “genetically recessive characteristics” which were mainly found in the secondary centre of diversification. About a decade later, Harlan (1971) proposed the non-centric origin theory where he argued that domestication was done in a geographically diffuse manner over a protracted period of time. Harlan argued that African rice and other crops such as sorghum and pearl millet underwent gradual and parallel domestication in different regions and therefore had multiple origins. Based on his theory, African rice domestication followed a protracted transition model as opposed to a rapid transition model (Allaby et al. 2008; Fuller 2007; Meyer et al. 2016; Purugganan 2019). The domestication process was associated with a long decline in effective population size for both the domesticate and progenitor which could have possibly been due to a long period of pre-domestication management (Meyer et al. 2016). It is postulated that the domestication of African rice was triggered by the depletion of wild rice populations which was attributed to the emergence of unfavourable climatic conditions in the Sahara (Cubry et al. 2018).

Genomics has great potential in tracing the origin, cultural and demographic history of cultivated species. In addition to giving valuable insights on domestication, DNA sequencing data is providing information on evolutionary events that preceded domestication. Results of various whole genome-based population genomic studies have pointed to a single origin thus supporting Porteres’s theory (Cubry et al. 2018; Veltman et al. 2019; Wang et al. 2014). Similar findings had earlier been made in a multi-locus nuclear gene sequence analysis (Li et al. 2011). The population level molecular analysis conducted by Wang et al. (2014), where whole genome data for a total of 114 African rice and *O. barthii* were analysed, marked the first attempt to map the domestication centre using whole genome sequences. This study which was the first to sequence the African rice genome structured the *O. barthii* population into 5 genetic groups. Out of these, one sub population designated as OB-V, was identified as the progenitor population based on its close genetic relationship with the domesticated species. However, this is sharply disputed by Choi et al. (2019) who analyzed whole genome sequence data for about 280 samples of African rice and *O. barthii*, including those studied by Wang et al., (2014). Ancestry analysis conducted by Choi et al. (2019) found that the progenitor population identified by Wang and his co-workers had high proportions of African rice ancestry. Moreover, this population possessed domestication mutations that are typically found in domesticated species. These authors concluded that the hypothesized progenitor population may have undergone significant hybridization with African rice or the samples may have been misidentified. This suggested that the study had major flaws that inevitably may have led to erroneous findings and conclusions. This problem of determining the direction of gene flow between wild and domesticated populations has plagued many domestication studies. For example, recent analysis of sequence data has supported the view that wild barley in Tibet originated by escape form domesticated populations (Zeng et al. 2018).

Analysis of whole genome sequences of *O. glaberrima* and *O. barthii* by Cubry et al. (2018) suggested that the exact domestication centre was in the Inland Niger Delta and specifically Northern Mali. The identification of the Inland Niger Delta as the cradle of African rice is consistent with linguistic, ethno botanic and archaeological evidence (Murray 2004; Portères 1966). Another whole genome based study by Veltman et al. (2019) found evidence seemingly supporting both the centric and non-centric theories and the authors seem to have been torn on which of the two to finally support. These authors observed a population structure where African rice samples collected from the proposed centre of origin in the Inland River Delta formed one genetic cluster. These *O. glaberrima* samples were more genetically diverse and had undergone less genetic differentiation from *O. barthii* than Coastal *O. glaberrima* samples. These findings suggest that domestication took place in the Inland areas before African rice migrated westward where the coastal samples were dispersed, consistent with Porteres theory.

Analysis of both nuclear and chloroplast genomes identified hot spots of rare alleles in the Inner Niger Delta region (Veltman et al.^ 2018^; Cubry et al. 2018) suggesting it may have been the ancestral site. The distribution pattern of rare alleles observed by these 2 studies seems to lend support to the centric theory. On the other hand, Veltman et al. (2019) reported the presence of ancestral variation and existence of multiple gene haplotypes in different sub-populations of African rice. Closely related findings were made by Choi et al. (2019) who reported the presence of domestication causal mutations in different geographical regions. Both of these latest genomic studies argued that these genetic occurrences may be a product of a parallel evolutionary process and not a single event. They suggested that this was enough evidence to support the non-centric model. These studies finally concluded that the single origin theory was incorrect or seriously flawed and therefore argued that the non-centric hypothesis provided a more credible model to explain the origin and domestication history of this cultivated *Oryza* species. Veltman et al. (2019) seems to question and discount the centric origin theory partly on the basis that African rice accessions did not form a monophyletic clade. This argument seems flawed as it has been shown that multiple origin crops have more chances of forming a monophyletic clade than those from single origins (Allaby et al. 2008). Moreover, even in cases of centric origin, post-domestication geographic based adaptation can lead to a polyphyletic clustering pattern. Despite the great advances in genomics witnessed to-date, it is clear that studies continue to report contradictory findings dimming hope that these advances offer an opportunity to conclusively settle debate on the origin and domestication history of African rice.

### African Rice Progenitor

**Fig. 1:**
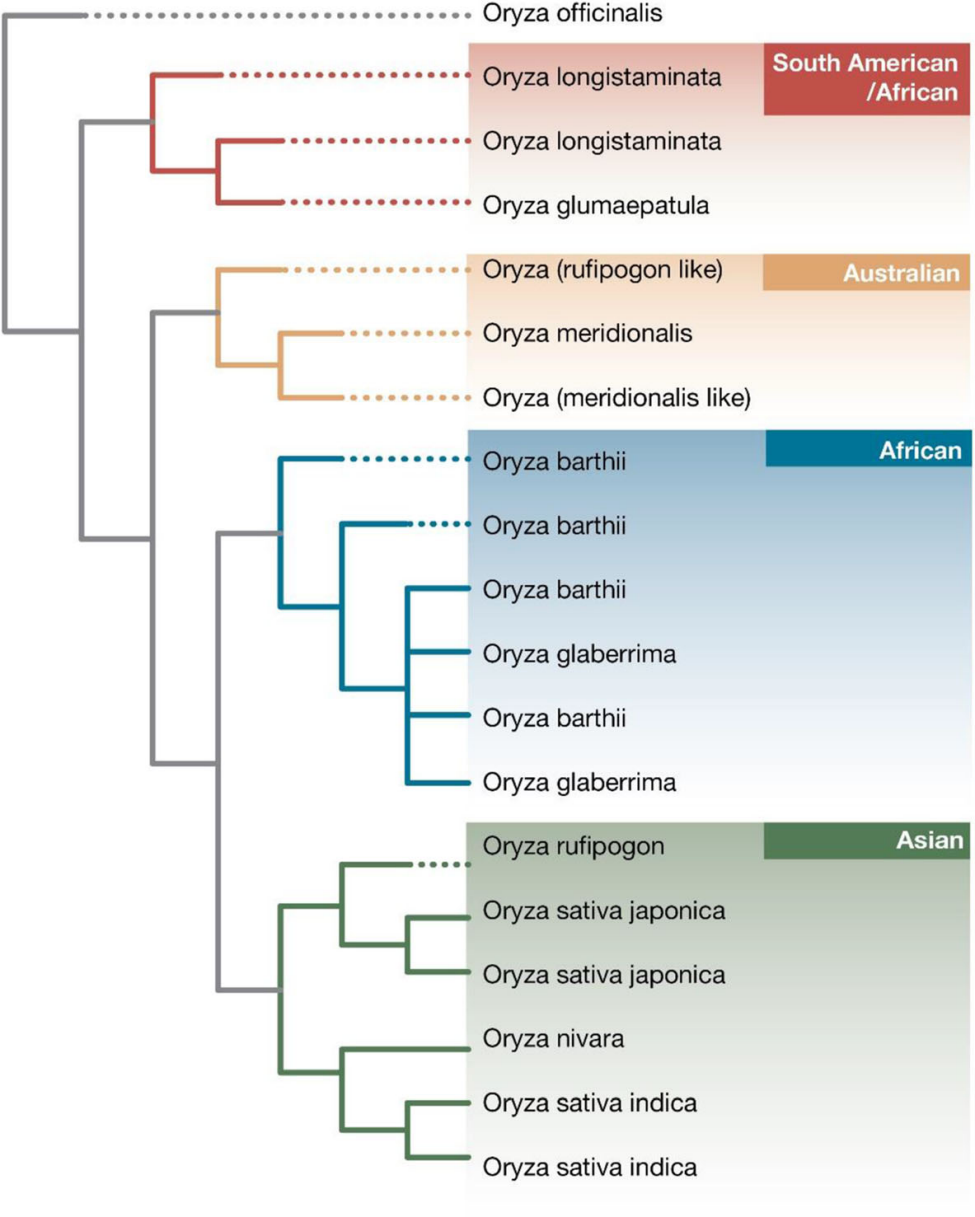
Phylogenetic relationships between various Oryza AA genome group species based on whole chloroplast genome sequences. Shows the close genetic relationship between *O. glaberrima* and *O. barthii*

Although there has been a few controversies on the origin and domestication history of African rice as already highlighted, there is almost complete consensus that the progenitor of African rice is *O. barthii*. Many studies, some based on whole genome nuclear and chloroplast data, have reported a very close morphological and genetic relationship between *O. barthii* and African rice (Fig. 1) (Duan et al. 2007; Huang et al. 2015; Stein et al. 2018; Wambugu et al. 2015; Wang et al. 2014). This suggests that these two species have a shared ancestry. Analysis of the genomes of these two species indicate that most of the polymorphic sites in their genomes are shared (Veltman et al. 2019). The sharing of some useful traits between the two species such as tolerance to biotic and abiotic stresses as well as unique starch traits may also be a sign of their close relationship (Wambugu et al. 2018). Owing to their close morphological similarities, it is difficult to distinguish *O. barthii* from the cultivated species particularly at the vegetative stage (Nayar 2014).

Due to relatedness between these taxa and others in the *Oryza* AA genome group, cases of species misidentification have been reported (Ndjiondjop et al. 2018b; Orjuela et al. 2014). In an effort at addressing the problem of species misidentification among African *Oryza* species including Asian rice, Ndjiondjop et al. (2018b) developed 332 species diagnostic markers. However, none of the markers could discriminate between *O. barthii* and African rice further indicating their genetic close relatedness. Despite the near unanimity, there has been a few opposing hypothesis on the progenitor of African rice that have been put forward. For over four decades one author has repeatedly argued that African rice originated from Asian rice through sympatric speciation (Nayar 1973, 2010, 2012, 2014). The two cultivated species have however been found to have great genetic differentiation (Huang et al. 2015) thus raising doubts on the validity of this hypothesis. Moreover, it is believed that African rice was domesticated before the introduction of Asian rice in Africa (Sweeney and McCouch 2007). This contradictory theory has received little attention as clearly its arguments seem less convincing.

### Genetic Diversity of African Rice and its Progenitor

The domestication process plays a key role in shaping the genetic variation in domesticated crops. It is typically associated with a loss of genetic diversity due to the domestication bottlenecks involved. Studies employing diverse approaches among them transcriptome sequencing (Nabholz et al. 2014), whole genome population analysis (Veltman et al. 2019; Wang et al. 2014), targeted gene sequencing (Li et al. 2011) and SNP genotyping (Ndjiondjop et al. 2017; Orjuela et al. 2014) have consistently reported extremely low genetic variation in African rice. Some studies have suggested that the loss of genetic diversity in African rice was unusually severe compared to other crops (Li et al. 2011). It has been described as the least diverse crop species ever documented (Nabholz et al. 2014). African rice contains about 24%–54% of the diversity found in its putative progenitor (Li et al. 2011). On the other hand, *O. sativa* sub species *Indica* and *Japonica* are reported to have lost more variation, with the reduction in genetic variation estimated to be about 80% and 90% respectively (Zhu et al. 2007). The assertion of a greatly unusual loss of genetic diversity in African rice doesn’t therefore seem to be entirely correct. Studies based on a small number of samples may underestimate levels of nucleotide diversity (Ndjiondjop et al. 2019) thereby leading to erroneous conclusions. Despite losing substantial genetic diversity during domestication, it still retained important traits such as weed competitiveness, tolerance to drought, resistance to African gall midge, rice mottle virus, stem borer, nematodes as well as adaptation to acidic and low phosphorus soils among others (Wambugu et al. 2013). A low diversity within *O. glaberrima* is not inconsistent with this taxa being an important source of novel divergent alleles relative to those in the *O. sativa* gene pool. These traits have been highly beneficial to rice breeding.

The low genetic diversity in African rice may have been caused by a narrow genetic diversity in *O. barthii* (Li et al. 2011) relative to that in the progenitors of Asian rice. However, it could also be due to a severe domestication bottleneck. This severe bottleneck has however not been detected in *O. barthii* (Meyer et al. 2016). Vaughan et al. (2008) reported that the low genetic variation was the product of a double evolutionary bottleneck that African rice went through; the first one being the divergence of *O. barthii* from its Asian ancestors and the second being the domestication process. Similar domestication bottlenecks have been reported for other crop species (Caicedo et al. 2007; Eyre-Walker et al. 1998; Hyten et al. 2006; Tenaillon et al. 2004; Zhu et al. 2007). It is also puzzling how a species with such low genetic variation can possess such wide tolerance to diverse biotic and abiotic stresses as has been recorded for African rice. This phenomenon may however be explained by the amount of neutral and functional diversity present in a taxa. Yıldırım et al. (2018) reported negative correlation between neutral and functional genetic diversity. Neutral diversity does not confer any adaptive potential to a taxa or population. It is therefore possible that African rice has relatively higher levels of functional diversity compared to neutral diversity. However, there is need for further research in order to gather empirical evidence on the amount of functional and neutral genetic diversity in African rice and whether there exists any relationship between these two forms of genetic diversity*.* Conscious or unconscious selection during domestication may have led to loss of useful variation. An example of this, as will be highlighted later, is seed size in African rice where mutations led to smaller seeds than those of the progenitor (Wu et al. 2017). Similarly, disease resistance genes form part of the potentially useful variation lost from African and Asian rice during domestication (Sakai and Itoh 2010; Zhang et al. 2014). Most of the important genes lost from the cultivated species are still present in wild AA genome species. With the increased understanding of the genetic basis of domestication traits, it is now possible to do a well-targeted reintroduction of this variation.

### Population Structure of African Rice

Knowledge of population structure provides valuable insights on the evolutionary and demographic history of domesticated crop species. The existence of strong population structure could however frustrate studies on crop demographies (Snodgrass and Hufford 2018). The nature and extent of population structure in African rice varies between studies and ranges from no or almost no existence of population structure (Huang et al. 2015; Li et al. 2011; Nabholz et al. 2014), to a population differentiation showing up to 5 sub-populations (Ndjiondjop et al. 2017; Orjuela et al. 2014; Semon et al. 2005; Veltman et al. 2019). Results of genetic studies provide no clear picture of the basis of the observed population differentiation, with some indicating it is linked to country of origin (Ndjiondjop et al. 2017; Ndjiondjop et al. 2019), phenotypes (Semon et al. 2005) and geography (Meyer et al. 2016; Orjuela et al. 2014; Veltman et al. 2019).

Analysis of population structure may provide information on whether the observed genetic structure is due to geographical based isolation or human mediated selection processes. This can potentially shed light on the number of times a crop has been domesticated. Analysis of 14 unlinked nuclear genes in 40 samples of African rice and *O. barthii* detected no population structure in African rice suggesting a single origin of this cultivated species (Li et al., 2011). As already highlighted, Wang et al. (2014) reported strong population structure in *0. barthii*. In this population genomic study, almost all the *O. glaberrima* accessions clustered with one of *O. barthii* genetic groups. This suggests a single origin of African rice in the area around Guinea, Senegal, Gambia, and Sierra Leone. As already highlighted, the genetic identity of this *O. barthii* population has been questioned by Choi et al. (2019) suggesting that it appears to have been misidentified, and supporting the single origin theory. However, as already stated, a monophyletic clustering pattern does not necessarily support the single origin theory (Allaby et al. 2008).

### Domestication Traits and their Underlying Genetic Mechanisms

Domesticated plants are typically distinguished from their progenitors by a series of mainly morphological and physiological traits which have collectively been referred to as the “domestication syndrome” (Hammer 1984). The key domestication traits in rice include loss of seed shattering, plant architecture, seed size, reduction in seed dormancy, time to flowering and maturity, reduction in awn length, panicle architecture and seed hull and pericarp color (Table 1). After selecting for these primary domestication traits, humans embarked on diversification or improvement of the domesticate (Meyer and Purugganan 2013; Pickersgill 2018), by targeting traits that enhance its adaptability to the prevailing local conditions and culinary preferences.

**Table 1:** Key domestication genes in African rice

Gene/QTL/Loci	Trait	Putative function	Causative mutation	Reference
OgSh1	Seed shattering	YABBY transcription factor	Unclear	(Wang et al. 2014)
*GL4*	Seed size and seed shattering	Myb-like protein	C/T SNP in exon 1 leading to premature stop codon	(Wu et al. 2017)
*OgSh3*	Seed shattering	GT-1-like trihelix transcription factor	C/T SNP	(Win et al. 2017)
*SH5*	Seed shattering	BEL1-type homeobox gene	Not identified	(Cubry et al. 2018)
*OgSh4*	Seed shattering	Myb3 DNA binding domain protein	Unclear	(Wang et al. 2014)
*RICE PLANT ARCHITECTURE DOMESTICATION* (RPAD)	Plant architecture	Zinc finger genes	113-kb deletion	(Wu et al. 2018)
*Prostrate growth 7* (*PROG7)*	Plant architecture	Zinc-finger transcription factor	Mutations in promoter region	(Hu et al. 2018)
*Regulator of Awn Elongation gene 3 (RAE 3)*	Awn formation		Not identified	(Furuta et al. 2015)
*Early heading date 1 (Ehd1)*	Heading date	B-type response regulator	Not identified	(Doi et al. 2004; Doi et al. 1998)
*Rc gene*	Pericarp colour	Regulatory protein in the proanthocyanidin synthesis pathway	Novel point mutation in exon 7	(Gross et al. 2010)
*Prostrate growth 1* (*PROG1)*	Plant architecture		Entire gene is absent	(Cubry et al. 2018)

Characterization of domestication genes is providing a wealth of information on the domestication process. Candidate gene analysis, Quantitative Trait Loci (QTL) mapping and linkage analysis have played pivotal roles in studying genetic mechanisms underlying domestication traits. In African rice, these studies are being aided by the availability of extensive genetic resources conserved in genebanks (Ndjiondjop et al. 2018a; Wambugu et al. 2013), availability of various types of advanced mapping populations (Doi et al. 1997; Win et al. 2017; Wu et al. 2018) and diverse types of genomic resources including genome reference sequences (Monat et al. 2017; Sakai et al. 2011; Stein et al. 2018; Wang et al. 2014). Compared to Asian rice, relatively little is known about the molecular and genetic basis of domestication traits in African rice (Furuta et al. 2015; Hu et al. 2018; Win et al. 2017). Analysis of genomic regions with unusual genetic divergence between African rice and its progenitor has been used to identify candidate domestication genes and the causative mutations (Wang et al., 2014). With the increased availability of whole genome sequences, genome wide association studies and resequencing approaches are enabling even greater understanding of the genetic architecture of these traits.

Most of the causal mutations associated with domestication have been SNPs though copy number variations and indels have recently been reported to also have genetic control of various domestication phenotypes (Lye and Purugganan 2019; Meyer and Purugganan 2013). These have in most cases resulted in altering the expression of domestication genes or led to loss-of-function (Meyer and Purugganan 2013; Wu et al. 2018). Indels were found to be enriched in putative domestication loci and to have higher levels of fixation in African rice compared to the progenitor, suggesting that they may have played a particularly useful role in the domestication of this taxa (Stein et al. 2018). In this section, we discuss the genetic basis of various domestications genes, well aware of the underlying difficulties in clearly differentiating traits selected during domestication and those targeted during the subsequent crop diversification stages. Some of the genes discussed below may have been targets of selection during the diversification or improvement phases that followed the domestication process (Chen et al. 2019; Takeda and Matsuoka 2008). Many of the domestication and diversification traits are genetically complex in nature, being under polygenic control (Huang et al. 2010; Yano 2001; Zhao et al. 2011). Genome wide analysis of African rice samples identified 37 selective sweeps in genes other than the known candidate domestication genes (Ndjiondjop et al. 2019), suggesting these may have been targets of post-domestication selection. On the other hand, selective sweeps were not detected in some of the known domestication genes (Veltman et al. 2019; Cubry et al. 2018) This raises the possibility that many of the genes underlying domestication and diversification traits are yet to be identified.

#### Loss of Seed Shattering

Seed shattering is a key adaptive trait for wild species as it helps them to perpetuate themselves by dispersing their offspring to surrounding areas and ecological niches (Purugganan and Fuller 2009). It is however highly undesirable for domesticated taxa as it leads to difficulty in seed harvesting and may result in yield loss. The transition from a shattering to a non-shattering phenotype therefore constitutes a major domestication trait whose selection has been documented in various plant families and taxa (Di Vittori et al. 2019). Loss of seed shattering is primarily attained through mutations or genetic mechanisms that disrupt the formation or proper functioning of the abscission layer. The key seed shattering genes identified in Asian rice include *sh4, qSH1, OsCPL1, OsSh1* and *SH5* (Ji et al. 2010; Konishi et al. 2006; Li et al. 2006; Lin et al. 2012; Zhou et al. 2012). Several orthologs of seed shattering genes in Asian rice have been found to control this trait In African rice. Some of the genes identified in African rice include *OgSh1* (Wang et al. 2014), *OgSh3/OgSh/GL4* (Win et al. 2017; Wu et al. 2017), *SH5* (Cubry et al. 2018) and *ObSH3* (Lv et al. 2018; Wang et al. 2014; Win et al. 2017; Wu et al. 2017) (Table 1). *GL4*, an ortholog of *SH4* in Asian rice, was found to be responsible for controlling seed shattering in African rice and to also have pleiotropic effects on grain length (Wu et al. 2017).

Several mutations have been identified as having a role in the genetic architecture of seed shattering in African rice. These include a C/T SNP in both *SH3* and *GL4* gene (Wu et al. 2017). The C/T SNP in *GL4* gene is however not fixed in African rice suggesting that there could be other genetic mechanisms regulating this phenotype. *ObSH3*, a YABBY transcription factor was identified as an additional genetic mechanism, with selection for a deletion present in this gene leading to loss of seed shattering (Lv et al. (2018). A double mutation in *SH3* and *GL4* has been found to lead to a variety of seed shattering phenotypes (Lv et al. 2018). Cubry et al. (2018) reported that *SH5* possessed signatures of human selection and identified a deletion in the coding region as the responsible mutation. This mutation is also present in *O. barthii* confirming the existence of additional genetic mechanisms controlling this phenotype in African rice. There appears to be some confusion regarding the nomenclature of the various seed shattering genes, specifically *SH3* and *SH4*. While (Li et al. 2006) indicated that *SH3* and *SH4* map to the same chromosomal location and are therefore the same (Cubry et al. 2018; Win et al. 2017), other studies consider them as different genes (Lv et al. 2018). The rice scientific community should make efforts to streamline this confused nomenclature by following existing guidelines and recommendations such as those of the Committee on Gene Symbolization Nomenclature and Linkage (CGSNL) (Choi et al. 2019). Clearly, the on-going advances in genomics have led to significant progress in the identification of mutations underlying various domestication phenotypes.

Despite the immense progress made in characterizing seed shattering genes, notable contradictions occur between studies as already highlighted. Comparative analysis of domestication candidate genes in *O. sativa, O. glaberrima* and *O. barthii* revealed that the ortholog of *OsSh1* is absent in African rice though interestingly, it is present in *O. barthii* (Wang et al. (2014). Instead of the *OsSh1* ortholog, a 45 kb deletion exists in African rice. However, *OgSh1* has been reported in some genomes of African rice and in all *O. barthii* genomes (Cubry et al. 2018). This therefore contradicts the earlier findings by Wang et al. (2014) which suggested the fixation of the *OgSh1* deletion in all African rice genomes*.* Similar analysis of *sh4* indicated that though the *O. sativa* causative mutation was absent in African rice, the promoter region of this gene possessed signatures of artificial selection suggesting that this region may have been the target of selection during domestication. The reduced or non-existent expression of this gene in African rice may have been due to this promoter region-targeted selection. These findings were however contradicted by (Cubry et al. 2018) who in addition to reporting the presence of the potential causal polymorphism in about 86% of the African rice samples, found no evidence of strong selection in the *sh4* gene or its promoter region. The findings by (Wang et al. 2014) were similarly contradicted by (Win et al. 2017) who reported that analysis of nucleotide diversity patterns suggested that the coding region may have been a target of selection but not the promoter region. These authors ruled out absent or reduced expression of *SH4* as being responsible for the non-shattering phenotype in African rice as suggested by (Wang et al. 2014). As will be highlighted later, the contradictory results obtained between key studies may perhaps be attributed to the nature and origin of the analysed samples as well as the data analysis method used. The variant alleles causing contradictions surrounding the fixation of mutations could be those ancestral haplotypes which could have persisted in some samples after domestication (Wu et al. 2017). Additionally, the observed partial sweeps could be due to mutations arising from selection localized in a certain culture or region (Meyer and Purugganan 2013). Geographic based isolation can hinder the movement of variant alleles thereby resulting in soft sweep rather than a hard sweep. The process of identifying true selective sweeps (Harris et al. 2018; Nielsen 2005) can be challenging due to various confounding effects such as demographic histories (Shah et al. 2020). While the current trend in population level whole genome based studies is to analyze many samples, wider sampling may still be necessary to address some of these challenges.

#### Plant Architecture

Plant architecture was a key target during the domestication process, with the main change being transition from prostrate to erect growth. The main differences between the two phenotypes is in the number of tillers and tiller angle, both of which significantly affect other agronomic traits. Through its potential role in enhancing photosynthetic efficiency and increasing planting density, this transition had a major impact on plant productivity, with the erect growth being associated with higher grain yields than prostrate growth (Huang et al. 2009; Wu et al. 2018). Genetic linkage analysis conducted by Hu et al. (2018) suggested that the erect or prostrate growth habit is controlled by a dominant gene. The gene was identified as *prostrate growth 7* (*PROG7),* a zinc-finger transcription factor gene which was found to be under strong positive selection. As will be highlighted here, in addition to *PROG7*, there could be additional genetic factors controlling plant architecture in African rice.

In Asian rice, the key gene responsible for plant architecture is *prostrate growth 1* (*PROG1)* which has been shown to have foot prints of strong positive selection (Huang et al. 2012; Tan et al. 2008)*.* This gene is however absent in the genome of African rice (Choi et al. 2019; Cubry et al. 2018; Monat et al. 2017) but present in the majority of *O. barthii* individuals (Choi et al. 2019; Cubry et al. 2018). Both of these later studies reported that the locus was under positive selection thus confirming its role in domestication. The deletion of *PROG1* was associated with erect phenotype in African rice and in all the *O. barthii* individuals possessing the deletion. On the other hand, *O. barthii* individuals possessing the *PROG1* gene exhibited prostrate plant architecture (Cubry et al. 2018). This clearly shows that in addition to *PROG7*, presence or absence of *PROG1* had some control on plant architecture in these two African *Oryza* species. In order to attain the erect phenotype, farmers seem to have selected for the loss of function mutations in *PROG1* and the *PROG1* deletion in Asian and African rice respectively. *PROG 7* gene of African rice seems identical to *PROG 1* gene of Asian rice (Hu et al. 2018). Wu et al. (2018) identified a 110 kb and 113 kb deletion next to the *PROG1* gene in Asian rice and *PROG1* deletion genomic region in African rice respectively which is also involved in regulating plant architecture. This deletion is located in the *RICE PLANT ARCHITECTURE DOMESTICATION* (*RPAD*) locus on chromosome 7 which contains a tandem repeat of zinc genes some of which have been shown to control plant architecture. A selective sweep spanning both *PROG 1* and the deletion in both cultivated species suggests that both were targets of human selection.

#### Seed Size

**Fig. 2:**
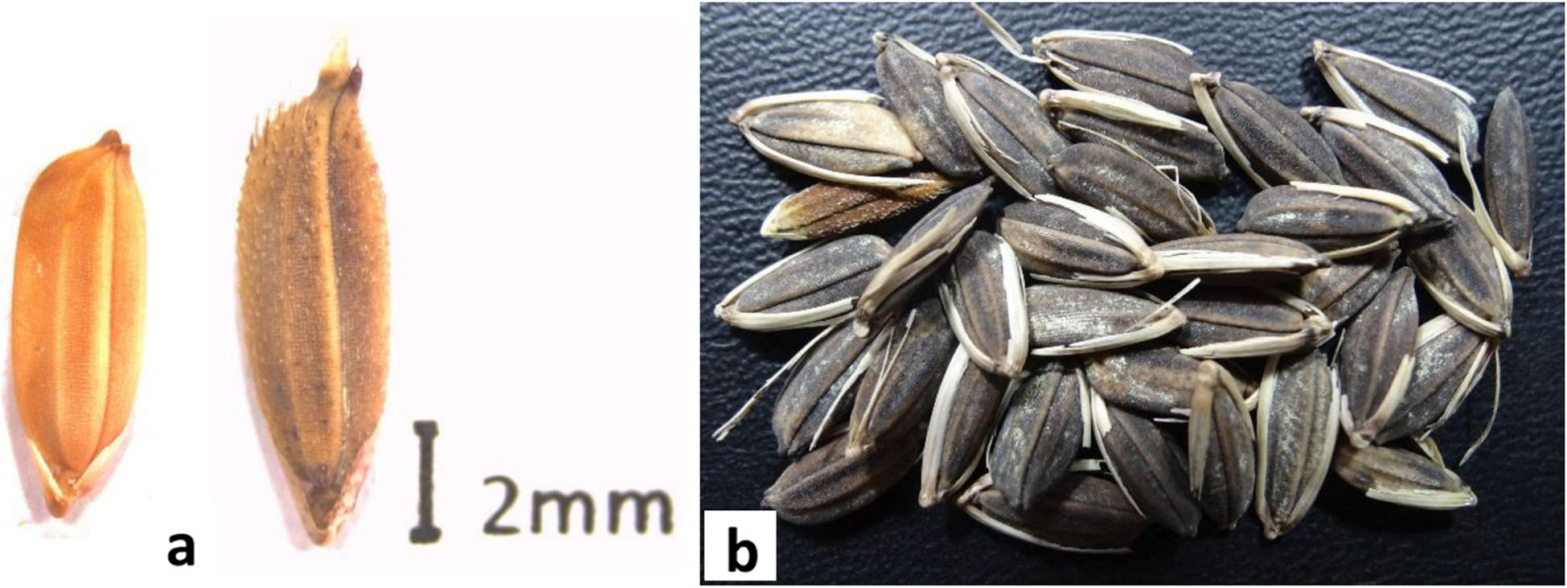
*O. glaberrima* and *O. barthii* seeds. **a** African rice exhibits an unusual domestication phenomenon where it has smaller seeds (left) than its putative progenitor (right) **b** Undehusked seeds of African rice

Seed size is a major determinant of plant yield and was therefore a key selection target during domestication of cereal crops (Fuller and Allaby 2009; Meyer and Purugganan 2013). Selection usually leaned towards larger seeds, with most domesticated species typically having larger seeds than their wild progenitors (Gegas et al. 2010; Han et al. 2015; Zuo and Li 2014). However, selection in African rice seems to have followed a different trajectory as it has smaller and shorter seeds than *O. barthii*, its putative progenitor (Fig. 2) (Katayama and Sumi 1995). Wu et al. (2017) dissected the genetic architecture of seed size in African rice and reported that it was controlled by *GL4* gene located on chromosome 4. Interestingly, *GL4* whose ortholog in Asian rice is *SH4*, has pleiotropic effects on both seed size and seed shattering. This study identified a C/T SNP in this gene as being responsible for the shift to smaller grains and loss of seed shattering. Since the African rice allele in this SNP has not been fixed, there is a likelihood that there exists additional genetic mechanisms controlling this trait. Genetic control of seed size in Asian rice has been found to be complex, with about 400 genes being reported to be involved in its regulation (Huang et al. 2013). Genetic mechanisms underlying seed size are conserved among many cereals (Tao et al. 2017). Such studies are providing useful insights on new genes to target during crop improvement. Manipulating the *GL4* loci in African rice by for example introducing the *O. sativa* allele will alter seed size thereby increasing seed yield and subsequently enhancing food security in the sub-Saharan region. Genes in wild populations may also be useful in enhancing seed size and yield in Asian rice (Henry 2019).

#### Pericarp Colour

Grain colour was a key target during the domestication of many cereal crops. In Asian rice, humans selected for the white (non-pigmented) grains over the red (pigmented) ones found in wild rice (Sweeney and McCouch 2007). Pericarp colour in Asian rice is controlled by the *Rc* gene, with two key mutations having been identified namely a 14 bp deletion and a point mutation which results in alleles designated as *rc* and *Rc-s* respectively (Gross et al. 2010). Several other mutations have been reported in Asian rice (Brooks et al. 2008). A comparative analysis of sequences at the *Rc* loci in African and Asian rice conducted by Gross et al. (2010), revealed that none of these mutations exist in African rice. This study identified a novel point mutation in exon 7 of the *Rc* gene in some white pericarp African rice samples which results in a premature stop codon. Some white and red pericarp African rice samples share the same sequences at the *Rc* gene suggesting that there could be other unidentified genetic mechanisms underlying this trait. The mutations in the *Rc* gene operate by disrupting the proanthocyanidin biosynthesis pathway thus resulting in pericarp pigmentation changes. With only seven African rice samples, the study by Gross et al. (2010) was quite limited in sampling and could therefore not provide conclusive information on these genetic mechanisms.

Compared to other domestication traits, pericarp colour remains less studied in African rice. For example, there seems to have been no effort made to investigate whether the *Rc* gene possesses signatures of human selection and hence a possible target during African rice domestication. However, due to the rarity of the white pericarp African rice genotypes, Gross et al. (2010) suggests that this trait may not have been preferred by farmers for selection during domestication. While selection in Asian rice has clearly been in favour of white rice, preference for either red or white pericarp African rice varieties varies between communities/countries (Mokuwa et al. 2014). Although presence of the red pericarp has been reported as one of the undesirable traits that have continually led to the displacement of African rice by Asian rice in West Africa, red pericarp African rice is preferred in some communities as it provides more satiety than white pericarp rice (Nuijten et al. 2009). Farmers have reported mixing the two types of rice in order to end up with a rice meal that provides greater satiety.

#### Loss or Reduction in Awn Length

The awn is an important morphological feature found in most grasses and wild relatives of most cereal crops. It plays an important role in seed dispersal as it helps the seed to attach to the fur of animals. In some cases such as barley, they also play a role in photosynthesis and therefore contribute to grain yield (Johnson et al. 1975). However, despite this important role, awns are undesirable in domesticated crops as they cause difficulties in seed harvesting. They were therefore a target of selection during domestication of rice and other cereal crops (Furuta et al. 2015; Luo et al. 2013). Awn formation in *O. sativa* is controlled by two genes namely *Regulator of Awn Elongation 1 (RAE1)* and *RAE2 (*Furuta et al. 2015*). RAE1* was found to be identical to *An-1* which had been reported earlier as being responsible for regulating awn formation, grain size and grain number (Luo et al. 2013). Loss of function in these two genes leads to the awnless phenotype. Despite these two genes being functional in *O. glaberrima*, they had no role in regulating awn development (Bessho-Uehara et al. 2016). This role is played by a third *Regulator of Awn Elongation gene* designated as *RAE3* which is located on chromosome 6 (Furuta et al. 2015). Many other genes have been reported as playing a part in awn formation in rice species but their role in African rice is still not yet known (Yoshimura et al. 2010), suggesting a gap in understanding the complex regulatory network of this trait. A candidate gene analysis is recommended in order to obtain insights on these genetic factors. Awn formation is a classic example of selection for the same phenotype by targeting different genes.

#### Heading Date

Flowering time is an important trait that determines the adaptability of a species in a certain geographical region. Although many studies classify variation in flowering time as a domestication gene (Doebley et al. 2006), others believe it was selected by humans as they tried to adapt domesticated crops into new production environments (Meyer and Purugganan 2013). Flowering time in most cereals is controlled by a major QTL, *Heading Date 1 (HD1)* (Liu et al. 2015). This gene has however been found to be missing from some genomes of African rice (Sanyal et al. 2010; Wang et al. 2014) and present in others (Monat et al. 2017). Flowering time in African rice has been found to be regulated by the *Early heading date 1 (Ehd1)* gene (Doi et al. 1998), with the African rice allele being dominant and confers early flowering (Doi and Yosimura 1998). This gene has been found to promote flowering under short day conditions in cases where there exists no functional allele of *HD1* (Doi et al. 2004) like in the case of African rice. In Asian rice, several other genes among them *Ghd7, Ghd8* and *OsPRR37* are known to regulate flowering date, with some having pleiotropic effects on other traits such as yield and plant height (Zhang et al. 2019). Not much is however known about these genes in African rice.

### Parallel and Independent Domestication of African and Asian Rice

Knowledge on the molecular basis of domestication traits is providing valuable insights on the parallelism of domestication between crops (Olsen and Wendel 2013; Purugganan 2014). Many studies have demonstrated that different cultures in Asia and Africa conducted parallel selection for the same traits and set of genes in a convergent but independent domestication of the two cultivated rice species (Cubry et al. 2018; Wang et al. 2014; Win et al. 2017). There has been a debate on whether the two phylogenetically close cultivated *Oryza* species followed identical or different domestication trajectory. Two scenarios have been found to have been at play. In the first scenario, the two species acquired the same phenotypes through selection of a common set of genes but in most cases the underlying mutations are independent. This has been observed for seed shattering where *sh1* is found to be conserved in both species although, as already highlighted, other genes are responsible for controlling this trait (Li and Olsen 2016). Genetic control for pericarp colour at the *Rc* gene is shared between Asian rice and some genotypes of African rice, though with independent mutations (Gross et al. 2010). The *RICE PLANT ARCHITECTURE DOMESTICATION* (*RPAD*) locus has been found to control plant architecture in both species (Wu et al. 2018). Similarly, there was convergent selection for the deletion and loss of mutation function at the *PROG 1* gene in African and Asian rice respectively (Cubry et al. 2018). In other cases, although seemingly less common, sharing of the same derived traits between the two lineages was through selection of different genes. Awn development presents a good example of this type of genetic control of parallel evolution of the same trait (Furuta et al. 2015). Indicative of convergent selection, studies have reported many loci possessing signatures of selection in both Asian and African rice (Huang et al. 2015). The parallel evolution between the two rice species can be used as a model to study whether and to what extent genetic insights obtained from one species can be applied on the other.

There has recently been growing evidence of phenotypic convergence of the same traits across different crop lineages (Pickersgill 2018; Rosenblum et al. 2014). The genetic and developmental basis of seed shattering at the *Sh1* locus is for example shared in maize, Asian rice, sorghum, African rice and foxtail millet (Jia et al. 2013; Li and Olsen 2016; Lin et al. 2012; Wang et al. 2014). The genetic mechanisms underlying heading date, specifically *HD1* is also conserved between rice, sorghum and foxtail millet (Liu et al. 2015). In addition to controlling plant architecture in the two domesticated rice species as already highlighted, the RPAD locus is also involved in regulating plant growth during the domestication of foxtail millet (*Setaria italica*) from its presumed progenitor, green foxtail (*S. viridis*) (Wu et al. 2018). There was also parallel selection for seed size in various cereals by targeting the same genes (Tao et al. 2017). These examples illustrate cases of parallel evolution of important traits particularly in the Poaceae family. Increased knowledge on parallel evolution has provided useful insights that will aid crop improvement by identifying the loci to target for the transfer of important traits between species.

### Challenges in Unravelling the Origin and Domestication History of African Rice

#### Altered Genetic Makeup of Study Populations

Most of the genetic resources that are found in ex situ and in situ conservation were collected in areas that have been disturbed through human activity and may have lost their original genetic makeup. Significant introgressions has for example been reported between African AA *Oryza* genome species and the South American AA species *O. glumaepatula* (Stein et al. 2018). Gene flow which has been found to confound patterns of rare alleles which are useful in mapping the domestication centre has been reported in African rice (Cubry et al. 2018). Genome wide data is providing greater resolution in identifying genotypes with mixed ancestry thereby helping avoid their confounding effects by dropping them in genetic studies (Choi et al. 2019). There has also been evidence of minor and largely unidirectional introgression from Asian rice, although this does not seem to have impacted any domestication loci (Huang et al. 2015). As stated earlier, cases of species misidentification are common in African rice (Choi et al. 2019; Martin et al. 1997; Ndjiondjop et al. 2018b; Orjuela et al. 2014; Wang et al. 1992). Wrong taxonomic identification could be caused by hybridization and the lack of clear distinguishing characters especially between the AA genome species. Feral weedy rice populations (Wedger and Olsen 2018) are common and may also confound genetic studies particularly if not properly identified. Despite its confounding effects, it is important to recognize the potential role that hybridization plays in generating novel diversity and enhancing the adaptive capacity of plant species.

The coincidence of African rice domestication with the southward expansion of the Sahara desert may have led to loss of important *O. barthii* populations and possible genetic drift in remaining populations (Cubry et al., 2018). Studies on rice evolution and domestication are further complicated by inadequate and biased sampling of genetic resources (Vaughan et al. 2008). Loss of important progenitor populations and any other germplasm that may enable proper, objective and unbiased study of evolution makes it difficult to accurately infer the domestication history of African rice. Whole genome data is enabling geneticists to overcome the challenge of phylogenetic discordance (Stein et al. 2018) which may be caused by use of misidentified samples or those that have lost their genetic integrity. It is now possible to study patterns of genetic admixture across the genome (Morrell et al. 2012). Similar to Asian rice (Londo et al. 2006), analysis of domestication history in African rice may also be complicated by large scale movement of people and widespread trade activities involving cultivated rice.

#### Data Analysis and Interpretation

The analytical tools used in studying domestication especially at the genome scale and the interpretation thereof may present significant challenges in accurately inferring the evolutionary and demographic history of cultivated species. Despite great advances in DNA sequencing and increased availability of whole genome data, analysis and interpretation of this data still remains a complicated and non-trivial exercise. Various technical and methodological challenges are now emerging particularly when dealing with large data sets (Cubry and Vigouroux 2019). Studies have continued to give contradictory findings and conclusions (Choi et al. 2019; Veltman et al. 2019; Wang et al. 2014). Some of these studies (Choi et al. 2019; Civáň et al. 2015; Huang and Han 2015; Huang et al. 2012; Wang et al. 2014) have analyzed the same datasets using different bioinformatics approaches. Such analysis has revealed diverse weakness ranging from various forms of analytical artefacts, flawed assumptions to use of samples whose genetic integrity may have been compromised.

After analyzing a total of 206 whole genome sequences of African rice and *O. barthii*, Veltman et al. (2019) did not detect any evidence of human selection in any of the candidate domestication genes. These authors suggested that this could be due to weaknesses in their analytical tools or could mean that these genes simply played no role in African rice domestication. However this latter suggestion does not seem feasible as many other studies (e.g. Wang et al. 2014) have found signatures of human selection in key domestication genes. Therefore, the most plausible explanation for the surprising findings by Veltman et al. (2019) is that the statistical approaches used and their assumptions may not have been very effective in identifying selective sweeps, a possibility that the authors clearly acknowledge and allude to. In somewhat related findings, Ndjiondjop et al. (2019) identified 37 candidate selective sweep regions in Africa rice but no candidate domestication genes were located within any of these regions. As already highlighted, identification of true selective sweeps may present significant challenges (Meyer and Purugganan 2013; Nielsen 2005). It therefore appears like the analysis of the massive amount of genome scale data that is currently being generated and the interpretation thereof may now emerge as a significant challenge. This is likely to further frustrate efforts aimed at resolving the contentious issues on the origin and domestication of both African and Asian rice.

### Future Prospects

It is expected that advances in genomics will continue reshaping our understanding of domestication. Epigenetics, archaeogenetics and genome editing are novel approaches that have potential for great application in domestication research (Schreiber et al. 2018).

## Data Availability

Not applicable.

## Abbreviations

QTL: Quantitative Trait Loci
RPAD: Rice Plant Architecture Domestication
HD: Heading Date
EHD: Early Heading Date
SNP: Single Nucleotide Polymorphism
RAE: Regulator of Awn Elongation
